# Iron modulates barrier integrity and stem cell function of small intestine during experimental colitis

**DOI:** 10.3389/fnut.2025.1545956

**Published:** 2025-05-09

**Authors:** Shubin Wang, Xiangjie Yang, Xiangjun Liu, Qin Wen, Lu Xu, Mei Feng, Jinyi Lang, Dengqun Liu

**Affiliations:** ^1^Radiation Oncology Key Laboratory of Sichuan Province, Sichuan Cancer Hospital and Institute, Sichuan Provincial Engineering Research Center for Tumor Organoids and Clinical Transformation, Sichuan Clinical Research Center for Cancer, Sichuan Cancer Center, School of Medicine, University of Electronic Science and Technology of China, Chengdu, China; ^2^Department of Medical Oncology, The Third People's Hospital of Sichuan Province, Chengdu, China; ^3^Department of Experimental Research, Sichuan Cancer Hospital and Institute, Sichuan Provincial Engineering Research Center for Tumor Organoids and Clinical Transformation, Sichuan Clinical Research Center for Cancer, Sichuan Cancer Center, School of Medicine, University of Electronic Science and Technology of China, Chengdu, China

**Keywords:** acute experimental colitis, iron, DFO, intestinal stem cell, organoid

## Abstract

**Background:**

Ulcerative colitis (UC) brings inconvenience to many patients with inflammatory bowel disease (IBD). Although colonic pathology is widely investigated, little attention has been paid to the disorders in small intestine of UC. In this study, we investigated the impairments of UC to small intestine and further explored how iron metabolism regulated epithelial integrity and the activity of intestinal stem cells (ISCs).

**Methods:**

Mice were treated by 2.5% dextran sulfate sodium (DSS) for 7 days to established acute experimental colitis. Small intestinal tissues were collected at different time points in the process of DSS-induced colitis. Histological analysis was used to evaluate the changes of small intestine, including H&E, Alcian blue and PAS staining, immunostaining, and qRT-PCR. Iron content was modulated by the supplementation of ferric citrate or depletion by deferoxamine (DFO). The influence of iron on the barrier integrity and stem cell function was further determined by histology, IEC-6 cell, and enteroid culture. ROS content was demonstrated by DHE staining. The proliferation of intestinal stem cells (ISCs) was shown by BrdU and Olfm4 staining, and Lgr5-tdTomato mice were used for lineage tracing study.

**Results:**

It was shown that during DSS-induced colitis, small intestine underwent a serious injury process, including dysregulated integrity and decreased proliferation of ISCs. Iron overload significantly exacerbated intestinal injury in tissues, epithelial cell line, and intestinal organoids. However, iron chelation by deferoxamine (DFO) would greatly suppress small intestinal injury. Mechanistically, iron overload exacerbated the generation of ROS and enhanced the infiltration of immune cells. In addition, STAT3 and ERK pathways in intestinal epithelium were impaired during experimental colitis, and iron content significantly interrupted the expression of p-STAT3 and p-ERK1/2 within small intestine.

**Conclusion:**

In summary, this study proved that small intestine was also impaired in experimental colitis, and iron content could affect DSS-induced small intestinal damage and regeneration, indicating the strategy of iron supplementation in clinical practice needs to be more cautious and consider more factors.

## 1 Introduction

During the recent decades, inflammatory bowel disease (IBD), including Crohn's disease (CD) and ulcerative colitis (UC), is regarded as an emerging global burden from the aspect of its gradually rising incidence (1). IBD has a variety of extraintestinal manifestations, with the most common complication being anemia (2). IBD anemia is detected in up to 70% of inpatients and 20% of outpatients (3) and is thought to affect one-third of the IBD population (4). IBD-related anemia can be caused by multiple factors, but it is usually caused by iron deficiency anemia (IDA). Anemia in these patients is also caused by functional or absolute iron deficiency due to inadequate dietary intake of iron, decreased absorption, and blood loss (5, 6).

IBD attracts more attention in both clinical practice and laboratory research due to several reasons. For example, IBD has profound negative influences on the life quality of patients and causes great increase of health costs, and more importantly, it is related to increasing risks of potential colorectal cancer (CRC) (7–9). Among IBD, CD is considered with genetic background. Therefore, UC is more widely investigated in laboratory study (10). Dextran sulfate sodium (DSS)-induced experimental colitis is one of the most commonly utilized animal models to mimic ulcerative colitis (11, 12). DSS belongs to a sulfated polysaccharide, and it was first described for use in hamsters in 1985 (13) and subsequently reported for use in mice in 1990 (14). DSS-induced experimental colitis has great simplicity and high reproducibility, which is the important reason that why it attracts many interests of IBD researchers to explore the pathogenesis and countermeasures of human IBD (15). When animals are provided with access to drinking water supplemented with different doses of DSS, acute chemical induced toxicity and inflammation first appears in colonic epithelium, especially within the middle and distal colon, which might explain why azoxymethane (AOM)-DSS-induced colorectal cancer appears in the distal colons (16). The clinical symptoms of DSS-induced colitis contain weight loss, diarrhea, and rectal bleeding, which is consistent with human UC. These symptoms usually are quantified as disease activity index (DAI) to reflect the severity of colitis (17).

Although experimental colitis mainly impairs the colonic epithelium, it has been reported that DSS also induces pan-gastroenteritis and in rat and mice, including colon, gastric mucosal, and small intestine (18), and even cardiac disorder (19). Small intestine (SI) is one of the most important organs responsible for nutrient absorption and mucosal immunity, and it has been indicated that DSS-induced colitis would also cause morphological and biochemical changes extending to SI. Using DPIV knockout mice, Yazbeck et al. had shown that DSS-induced damage was not constrained to the colon but also extended to small intestine (20), and loss of DPIV activity resulted in functional adaptations to brush border enzyme activity. DPIV inhibitors are now a recognized therapy for type-II diabetes. Some researchers have characterized the effects of Lactobacillus fermentum BR11 on DSS-induced small intestinal damage, and they found increased ileal crypt depth and cell proliferation (21). Paneth cells constitute the niche of ISCs, and DSS would induce decrease of the quantity of Olfm4^+^ and Lgr5^+^ ISCs. Meanwhile, Lyz1^+^ Paneth cells would dedifferentiate into ISC-like cells and give rise to ISCs and other epithelial lineages to promote the regeneration of SI epithelium (22). Inflammatory process in the small intestine after DSS treatment is also associated with dysmotility and altered barrier permeability, and it is revealed that ileitis affects enteric dopaminergic neurotransmission, and dopaminergic pathways participate in IBD-mediated neuromuscular dysfunction (23).

Many nutrients affect the physiological function of small intestinal epithelium and the regeneration after DSS-induced injury. DSS declined the percentage of small intestinal intraepithelial lymphocyte (IEL) γδ-T cells and higher mRNA expressions of IFN-r, TNF-a, IL-17, and complement 5a receptor in γδ-T cells. Glutamine (Gln) increased the proportion of small intestinal IEL γδ-T cells and downregulated the expression of inflammatory mediators in them, which might alleviate the severity of small intestinal epithelial injury (24). Sunset yellow (SY), an azo dye, is commonly used in the food industry. In DSS-treated mice, DAI and expression levels of IL-1b and TNF-a were enhanced in the SY group, concluding that SY exacerbated DSS-induced intestinal inflammation. SY could disturb the homeostasis of the small intestinal epithelium by generating high levels of ER stress and oxidative stress, with long-term continuous consumption of SY potentially increasing the risk of intestinal inflammation (25). Therefore, inappropriate uptake of nutrients might impair the progression of DSS-induced small intestinal injury.

Iron deficiency anemia (IDA) is often present in patients with IBD, and the main source of excess iron loss is intestinal bleeding (26). Treatment of all patients with IDA with or without symptoms is recommended by correcting the underlying cause and supplementing adequate iron stores (4). However, there is a lack of research on whether additional iron supplementation improves IDA and causes additional damage to the small intestine. Among different nutrients, iron is mainly absorbed by small intestine. Iron is indispensable to the energy production, DNA synthesis, and cell proliferation. Sufficiency of iron is critical to maintain tissue homeostasis, but overload of iron might increase the level of reactive oxygen species (ROS), which exhibits cellular toxicity. Therefore, it is important to maintain the balance of iron metabolism for both physiological health and the prognosis of many diseases. For example, iron status and inflammation are strongly linked during the process of aging (27). Iron is also tightly linked to infection and immunity (28). Both invading pathogens and host cells require appropriate iron to exert their individual biological functions (29). During the inflammation status, appropriate ferritin levels represent an important host defense mechanism that deprives bacterial growth of iron and protects tissue homeostasis. Both anemia and hyperferritinemia are harmful to human health (30). Oxidative stress in acute lung inflammation also results iron accumulation in macrophages (31). Iron deposition was involved in the occurrence of pulmonary fibrosis in bleomycin (BLM)-treated mice and β-thalassemia-induced lung injury, and chelation of iron by deferoxamine (DFO) significantly reduced the degree of these lung injuries (32, 33).

In the previous study, we found iron depletion would greatly affect the onset and healing of DSS-induced colitis. Meanwhile, we observed that small intestine was also impaired by DSS-induced inflammation. In the present study, we aimed to determine the dynamic histological changes of small intestine in DSS-induced experimental colitis and explore how iron metabolism would modulate epithelial integrity, ISCs, and inflammation responses within small intestine. Our results implicated that the balance of iron might be an important intervening target during the prevention and treatment of human IBD.

## 2 Materials and methods

### 2.1 Mice

In this study, 6- to 8-week-old male C57BL/6 mice were ordered from HFK Bioscience Co., LTD (Beijing, China). B6.129P2-Lgr5^tm1(cre/ERT2)Cle^/J (Lgr5-EGFP-IRES-CreERT2) and B6;129S6-Gt(ROSA)26Sor^tm14(CAG − tdTomato)Hze^/J (tdTomato) mice were purchased from Jackson Laboratory (Bar Harbor, ME, USA), and they were crossed to generate Lgr5-tdTomato offspring. The genotype of their pups was determined using the protocols provided by Jackson Laboratory. Mice used in this study were kept in a specific pathogen-free (SPF) facility under a 12-h light/dark cycle with access to water and food *ad libitum*. Experimental treatments were in accordance with guidelines for Care and Use of Laboratory Animals, and the protocols were approved by the Ethics Committee of Sichuan Cancer Hospital & Institute (SCCHEC-04-2024-038).

### 2.2 Establishment of DSS-induced colitis and drug treatment

Mouse experimental colitis was induced as previously elsewhere. In brief, C57BL/6 mice or Lgr5-tdTomato mice were randomly divided into different groups and treated with free access to drinking water supplemented with 2.5% colitis grade dextran sodium sulfate (DSS) (#216011080, MW: 36,000–50,000, MP Biomedicals) for 7 consecutive days. Mice were simultaneously treated with ferric citrate (#44941, Sigma) by daily intraperitoneal (i.p) injection at the dose of 15 mg/kg (34). Some mice received deferoxamine mesylate (DFO) (HY-B0988, MCE) also by daily i.p injection at 50 mg/kg (35). The number of mice in each study was described in the relevant figure legend, and 4–6 mice were included for each time point of different treatments. Both iron and DFO were given from Day 1 to Day 7. The influences of drug treatment on blood iron level were determined by red blood cell (RBC) and hemoglobin levels using a Systemax blood analyzer.

### 2.3 Disease activity index (DAI) and FITC-dextran permeability

The severity of mouse colitis was evaluated by disease activity index (DAI), which was determined by grading of weight loss, stool consistency, and rectal bleeding. For details, weight loss was scored from 0 to 4, which was classified as none, 1–5%, 5–10%, 10–15%, and 15–20% weight loss. Stool consistency was also scored from 0 to 4, indicating normal stool, semi-normal stool, loose stool, loose stool adhered to anus, and liquid stools adhered to the anus. Rectal bleeding was determined as follows: 0 = normal; 1 = seminormal; 2 = positive hemoccult; 3 = blood traces in stool visible; and 4 = gross rectal bleeding. These three parameters lead to a maximum of 12 scores for DAI. The impact of iron modulation on intestinal permeability was evaluated by FITC-dextran experiments as previously described (36) with appropriate modification. In brief, at the end of DSS-induced colitis, mice were starved for 14 h and gavaged with 50 mg/kg FITC-dextran (FD4, 3,000–5,000, Sigma-Aldrich, USA) in a volume of 0.2 mL. Blood was collected after 4 h to prepare serum samples. The fluorescent intensity of FD4 in serum was measured by Cytation 5 (Bio-Tek, USA).

### 2.4 Sampling and histological staining

Gastrointestinal tract was collected at the indicated time as described, respectively. Mice were given i.p injection of 100 mg/kg BrdU at 90 min before tissue collection. Tissues were quickly removed and placed on ice. Gross images of intestines were captured. Different segments of small intestines, including duodenum, jejunum, ileum, were flushed with ice-cold PBS and fixed with 4% precooled paraformaldehyde (PFA) (#BL539A, Biosharp) for more than 48 h. Tissues were further trimmed, dehydrated, and paraffin-embedded using our standard protocols. Then, 4 μm thick sections were dewaxed and rehydrated for subsequent hematoxylin–eosin (H&E) staining regularly.

### 2.5 Alcian blue and PAS staining

Intestinal goblet cells were stained by Alcian blue and periodic acid-Schiff (PAS) staining. Tissue slides were processed regularly before staining. Alcian blue was stained for acid mucus polysaccharides with Alcian blue kit (Catalog: #E670107, BBI). PAS staining was used to stain neutral mucus substances by PAS Staining Kit (Catalog: G1281, Solarbio). Each step of the staining procedures was performed under the instruction protocols provided by the manufacturers. Nuclei were stained with fast red or hematoxylin solution.

### 2.6 Immunostaining

Small intestinal sections were routinely processed before staining. Antigen retrieval was performed for 20 min in boiling TRIS-EDTA Antigen Retrieval Solution (#BL618A, Biosharp) after tissue rehydration, and blocking was conducted with 1% bovine serum albumin (BSA) (#A7906, Sigma) solution, containing 0.5% Triton X-100. Primary antibodies were diluted at 1:200 and then incubated overnight at 4°C. The details of primary antibodies used in this study are shown in Supplementary Table S1. On the next day, primary antibodies were washed, and HRP-linked secondary antibody (ZSBio, Beijing, China) was incubated for 40 min. DAB kit (ZSBio, Beijing, China) was used for immunohistochemical (IHC) visualization. Hematoxylin was used for counterstaining to show nucleus. For immunofluorescent staining, Alexa Fluor™ 594-conjugated highly cross-adsorbed donkey anti-rabbit or mouse IgG (H+L) antibodies (Thermo Fisher, USA) were used, and nuclei were stained with DAPI (Vector Laboratories, Burlingame, CA). Slides were observed with Olympus BX53 and Nikon Confocal microscope.

### 2.7 Lineage tracing of Lgr5^+^ ISCs

To examine how iron metabolism influenced Lgr5^+^ ISCs, Lgr5-tdTomato mice were used in the next study as previously described (37). In brief, mice were treated with DSS in combination of ferric citrate or DFO for 7 continuous days. On day 6, mice were intraperitoneally injected with tamoxifen (2 mg/20 g) to induce the expression of Cre recombinase in Lgr5^+^ ISCs. These ISCs and progenies would be labeled by tdTomato. Intestines were collected at day 7. Small intestines were removed, flushed, and fixed for the preparation of frozen sections. Images were captured with Nikon Confocal microscope.

### 2.8 Cell culture

IEC-6 cell line was introduced from ATCC and kept in our laboratory. It was cultured following the standard protocol described by ATCC. In brief, cells were cultured in DMEM medium supplemented with 10% FBS and 1% penicillin/streptomycin solution. Cell culture was performed in the incubator (Thermo Fisher, USA) with 5% CO_2_ at 37°C. Cells were treated with LPS (100 ng/ml) or hydrogen peroxide (H_2_O_2_) (1 mM) to induce inflammation and oxidative stress. Ferric citrate (100 mM) and DFO (100 μM) were, respectively, loaded. Cell viability was determined by CCK-8 kit (Dojindo, China). For staining, IEC-6 cells were fixed with 4% PFA for 30 min, washed with cold PBS, and then stained for fluorescence using antibodies against ZO-1 or Occludin. The toxicity of iron and DFO on LPS-treated IEC-6 was determined using the lactate dehydrogenase (LDH) Cytotoxicity Assay Kit (Beyotime, Shanghai, China), following the manufacturer's protocol.

### 2.9 Culture of organoid

Small intestinal tissues were quickly isolated from healthy or colitis mice to culture enteroids. In brief, intestines were flushing with ice-cold PBS, cut into 3–5 mm pieces, vigorously washed in PBS to remove bacteria, and finally placed into 5 mM EDTA chelation buffer supplemented with 1% FBS. Tissues were immersed in ice bath for 30 min and then washed to remove EDTA. Crypts were released by vigorous shaking. Intestinal crypts were purified by filtration through 70 μm cell strainer. Crypts were counted and centrifuged at 4°C and 800 × g for 3 min, then mixed with Matrigel (#354230, Corning, USA), and finally seeded into 96-well fat-bottom plates to culture enteroids using IntestiCult™ Organoid Growth Medium (#06005, STEMCELL Technologies, Canada) supplemented with 100 μg/mL streptomycin and 100 units/mL penicillin. The growth of organoids was observed and compared with healthy organoids. Healthy enteroids were also treated by 100 mM ferric citrate and 100 μM DFO with or without LPS and H_2_O_2_. Cytotoxicity of iron and DFO on LPS-treated enteroids was also examined using LDH Cytotoxicity Assay Kit as mentioned above.

### 2.10 ROS detection

Reactive oxygen species (ROS) was examined by dihydroethidium (DHE) probe (S0063, Beyotime) according to the manufacture's protocol. In brief, DHE dissolved in DMSO was loaded into the culture medium at a concentration of 5 μM. Cells, crypts, or organoids were incubated with DHE probe at 37°C for 30 min and then observed using the appropriate microscope.

### 2.11 Image capture and data collection

Images of H&E and IHC staining of slides and organoids in culture plates were captured by M5000 (Thermo Fisher, USA), Cytation 5 (BioTek, USA), or BX53 (Olympus, Japan) microscope. IF staining images were captured by an A1R confocal microscope (Nikon, Japan) or Zeiss Axio Observer with Apotome3. All the images in the study were evaluated by two independent researchers, who were blinded to the specific information of the subjects to reduce the subjective bias of the investigators. Image parameters were analyzed by ImageJ (NIH, USA) for each image. Each value was calculated based on at least three independent replicates.

### 2.12 qRT-PCR assay

RNA was extracted from intestinal tissues or cultures by RNAiso (9109, TaKaRa, Japan), and RNA quality and concentration were calculated using NanoDrop2000 (Thermo Scientific). Reverse transcription (RT) was performed using Hifair II 1st Strand cDNA Synthesis SuperMix (11137ES60, YEASEN, China). Reaction system was prepared using Hieff qPCR SYBR Green Master Mix (11203ES08, YEASEN). Ad qPCR was performed on C1000 qPCR machine (Bio-Rad, USA). Primer sequences used in this study are listed in Supplementary Table S2. Gene expression results were normalized to that of β-actin, and the relative expression was determined by the 2^−ΔΔCt^ method.

### 2.13 Statistics

Data were presented by mean ± SD and analyzed using GraphPad Prism 9 (GraphPad Software, USA). Comparison between two different groups was conducted by two-tailed unpaired Student's *t*-test or non-parametric test depending on whether the data conformed to normal distribution. Comparison of multiple groups was analyzed using one-way analysis of variance (ANOVA) with *post-hoc* Tukey's test. *P* < 0.05 were considered as statistically significant different (^*^P < 0.05, ^**^P < 0.01, ^***^P < 0.001, ^****^P < 0.0001). A *P-value of* >0.05 was considered as non-significant (*n.s*).

## 3 Results

### 3.1 DSS-induced colitis impaired the histological characteristics of small intestine

**Figure 1:**
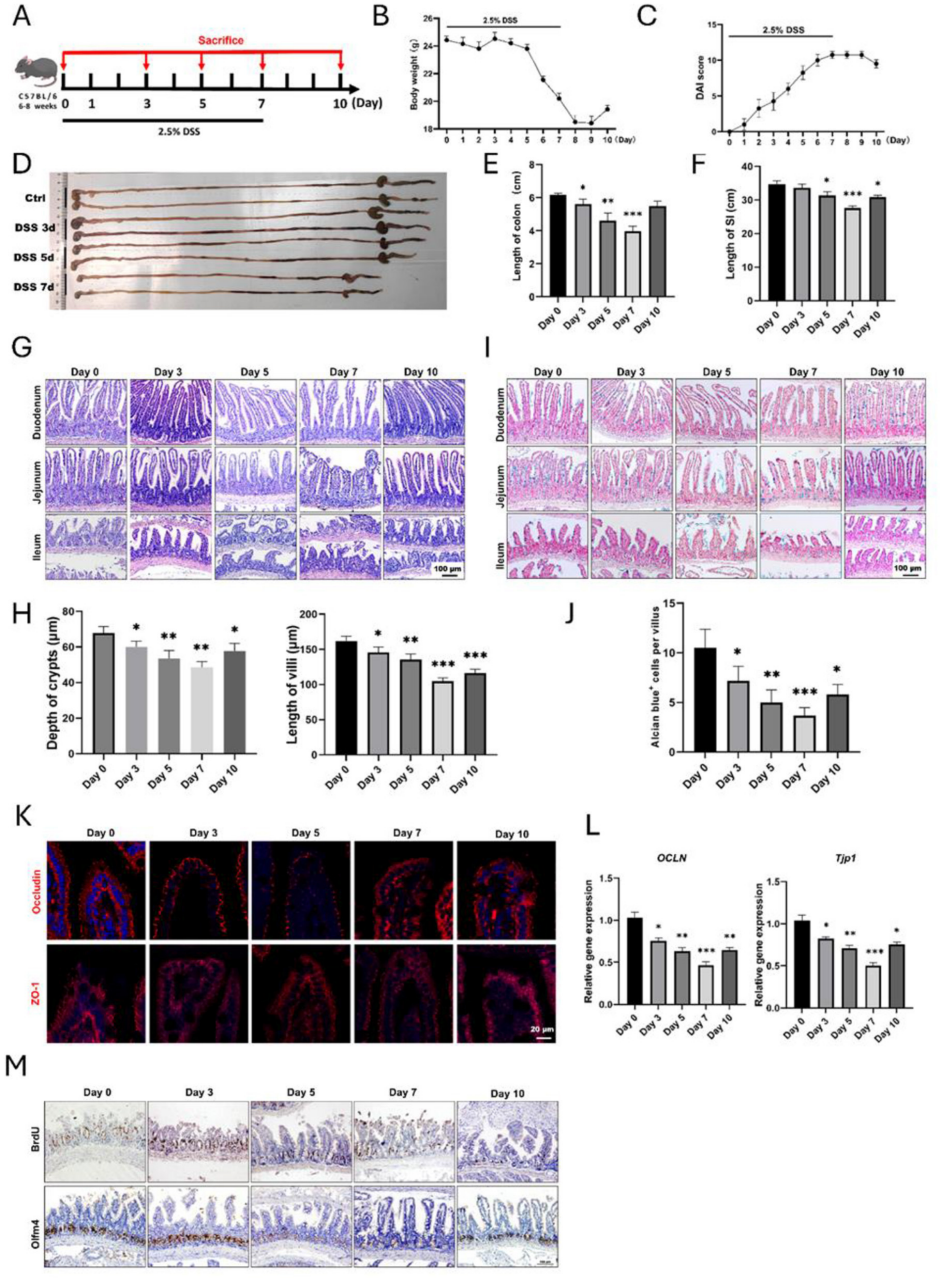
Histological changes of small intestine during DSS-induced colitis. **(A)** Schematic diagram for the establishment of experimental colitis using 2.5% DSS. **(B)** Body weight changes of mice after DSS treatment (*n* = 4 per group). **(C)** Disease activity index (DAI) score during the whole process of experimental colitis (*n* = 4 in each group). **(D)** The representative gross image of gastrointestinal tract at different time points. **(E)** Statistical analysis for the length of colons (*n* = 4 each point). **(F)** Analysis for the length of small intestines after DSS treatment, and four mice were included at each time point. **(G)** Representative H&E images for different segments of small intestine after DSS treatment. **(H)** Statistical analysis showed the changes in depth of crypts and length of villi of small intestine (*n* = 4 each point). **(I)** Alcian blue staining images of small intestine. **(J)** Quantitative data of goblet cells in the villous epithelium of ileum. **(K)** IF images showed the expression of Occludin and ZO-1 in small intestine. **(L)** Relative gene expression levels of *OCLN* and *TJP-1* in small intestine examined by qRT-PCR. **(M)** Representative IHC images of BrdU and Olfm4 after DSS-induced colitis. *P < 0.05, **P < 0.01, ***P < 0.001.

In this study, we first established experimental colitis with the supplementation of 2.5% DSS in drinking water (Figure 1A). Mice had access to food and water *ad libitum*. As expected, the body weights of mice were gradually declined with the drinking of DSS and regained after the withdrawal of DSS (Figure 1B). DAI could directly reflect the severity of colitis, and we observed a representative increasing of DAI score and a declining after withdrawal of DSS (Figure 1C), indicating a reliable experimental colitis was established. When we examined the gross appearance of gastrointestinal tract, it was found that the length of colons underwent decrease and recovery (Figures 1D, E). Interestingly, their small intestines were also experienced the same changes (Figures 1D, F), indicating that experimental colitis also caused apparent injury to small intestine. For example, the length of small intestine at day 7 was 27.61 ± 0.63 cm, which was significantly shorter than the control group (34.71 ± 1.01 cm). To confirm these results, we observed the histology of small intestine by H&E staining, and it was found that the intestinal epithelium was obviously damaged at day 5 and day 7, especially in the distal ileum (Figure 1G). Quantitative results showed significant decreasing the length of crypts and villi (Figure 1H). Subsequently, we used Alcian blue to stain goblet cells within the epithelium of small intestine, and it was shown that the number of goblet cells was declined after DSS treatment (Figures 1I, J). Moreover, the immunofluorescent staining and qPCR results for Occludin and ZO-1 identified that the epithelial integrity of small intestine was greatly impaired (Figures 1K, L). Importantly, using IHC staining, we found there were less BrdU^+^ proliferative cells and Olfm4^+^ ISCs within intestinal crypts caused by DSS treatment (Figure 1M). Therefore, these data supported that small intestine would also be apparently impaired due to DSS-induced inflammatory response.

### 3.2 Intervention of iron content affected DSS-induced injury of intestinal epithelium

**Figure 2:**
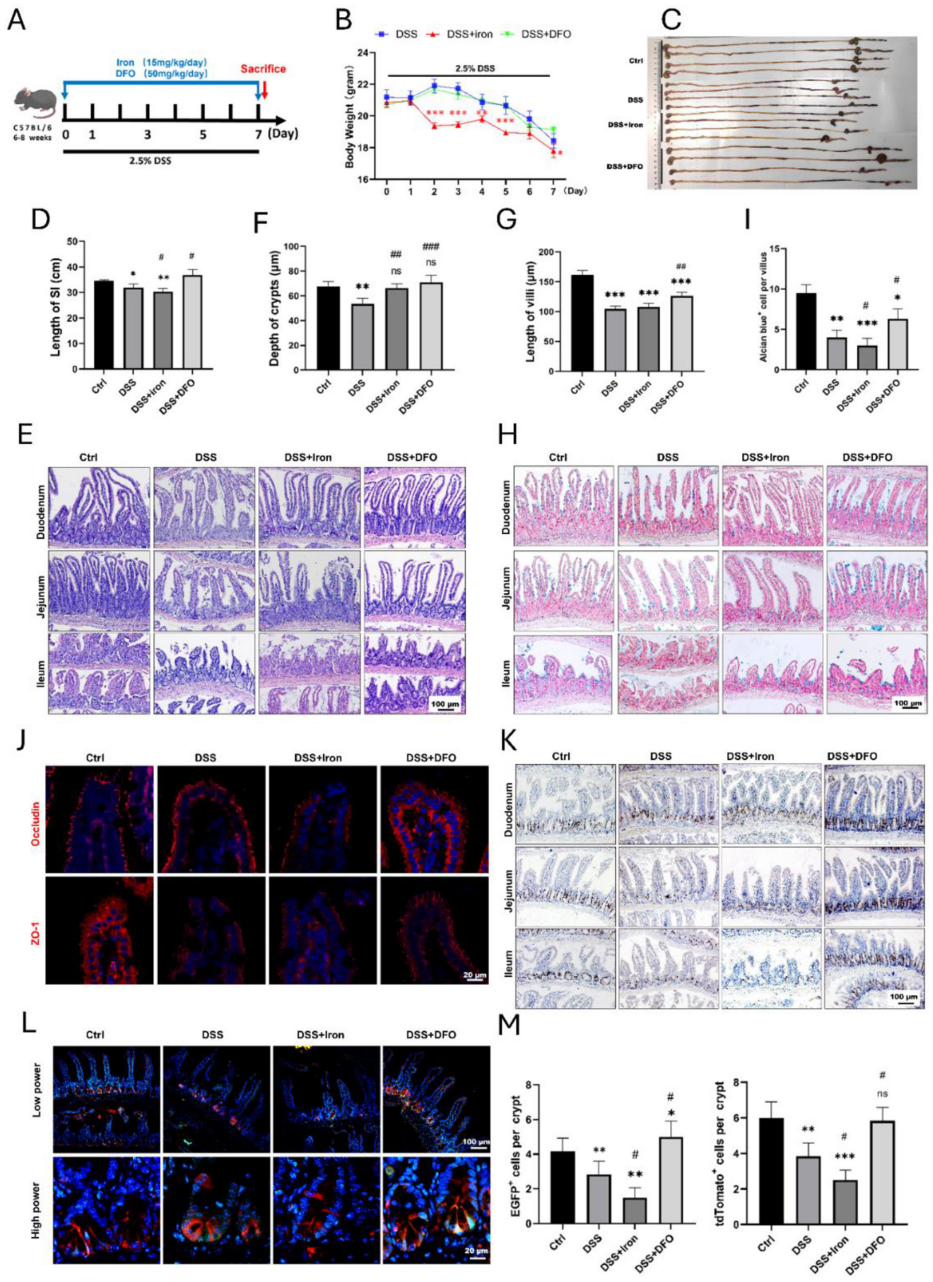
Iron modulation influenced epithelial injury of small intestine caused by DSS treatment. **(A)** Schematic diagram for the intervention of iron content during experimental colitis. **(B)** Weight loss of mice in different groups (*n* = 4 each group). **(C)** Representative images of the whole gastrointestinal tract in each group (*n* = 4 per group). **(D)** Statistical analysis for length of small intestine in each group. **(E)** Representative H&E images of small intestinal segments in different groups. **(F)** Analytic results for the depth of crypt in each group (*n* = 4 per group). **(G)** Statistical analysis of the length of villus in different groups (*n* = 4 each group). **(H)** Representative Alcian blue staining pictures. **(I)** Quantitative analysis for the number of goblet cells in ileal epithelium. **(J)** IF staining images of Occludin and ZO-1 staining in different groups. **(K)** Representative IHC images of BrdU in different groups. **(L)** Lineage tracing in Lgr5-EGFP; tdTomato mice after different treatments. **(M)** Statistical analysis of the EGFP positive Lgr 5^+^ ISCs and tdTomato^+^ progenies in different groups. *Compared to control group, ^#^compared to DSS group. **P < 0.01; ***P < 0.001; ^*##*^*P* < 0.01; ^*###*^*P* < 0.001.

Iron is an inevitable element for the maintenance of tissue homeostasis and inflammatory process, so we determined how intervention of iron levels would impact small intestinal injury during DSS-induced colitis. In brief, mice were treated by intraperitoneal (i.p) injection of ferric citrate at the dose of 15 mg/kg or DFO at 50 mg/kg during the period of DSS-induced colitis (Figure 2A). The body weight of DSS-treated mice had an obvious decrease as expected. Meanwhile, the supplementation of iron exaggerated the loss of body weight compared with DSS group, and iron depletion by DFO greatly alleviated the body weight loss (Figure 2B). The overview of abdominal cavity showed different appearance of gastrointestinal tract. There were more luminal blood contents in small intestine of iron treated mice, which was better alleviated in DFO-treated group (Supplementary Figure S1A). We found the lengths of small intestines were also different in each group, iron overload resulted in shorter length, and DFO group had longer small intestines (Figures 2C, D). Iron supplementation caused more serious damage to intestinal epithelium during DSS treatment. Similarly, mice in DFO group had better histological characteristics (Figures 2E–G). Mucus-secreting goblet cells were stained by Alcian blue and PAS, and there were less goblet cells in iron-treated DSS mice, but DFO rescued such a decrease (Figures 2H, I, Supplementary Figure S1B). Immunostaining and qPCR results showed iron supplementation led to more serious downregulation of both Occludin and ZO-1, and DFO reversed these effects (Figure 2J, Supplementary Figure S1C). Moreover, we found iron treatment exacerbated the decreasing of BrdU^+^ proliferative cells (Figure 1K) and mRNA level of Mki67 gene (Supplementary Figure S1D). qPCR showed iron promoted the proinflammatory factors, such as IL-1b and TNF-a, and decreased anti-inflammatory factor IL-10 (Supplementary Figure S1E). Importantly, lineage tracing study revealed that iron overload injured EGFP-Lgr5+ ISCs and inhibited their differentiation, and DFO alleviated DSS-induced injury (Figures 2L, M). Collectively, these data supported that intervention of the intestinal iron content could influence the intestinal injury caused by DSS-induced inflammation.

### 3.3 Iron overload delayed the regeneration of intestinal epithelium and promoted fibrosis after withdrawal of DSS

**Figure 3:**
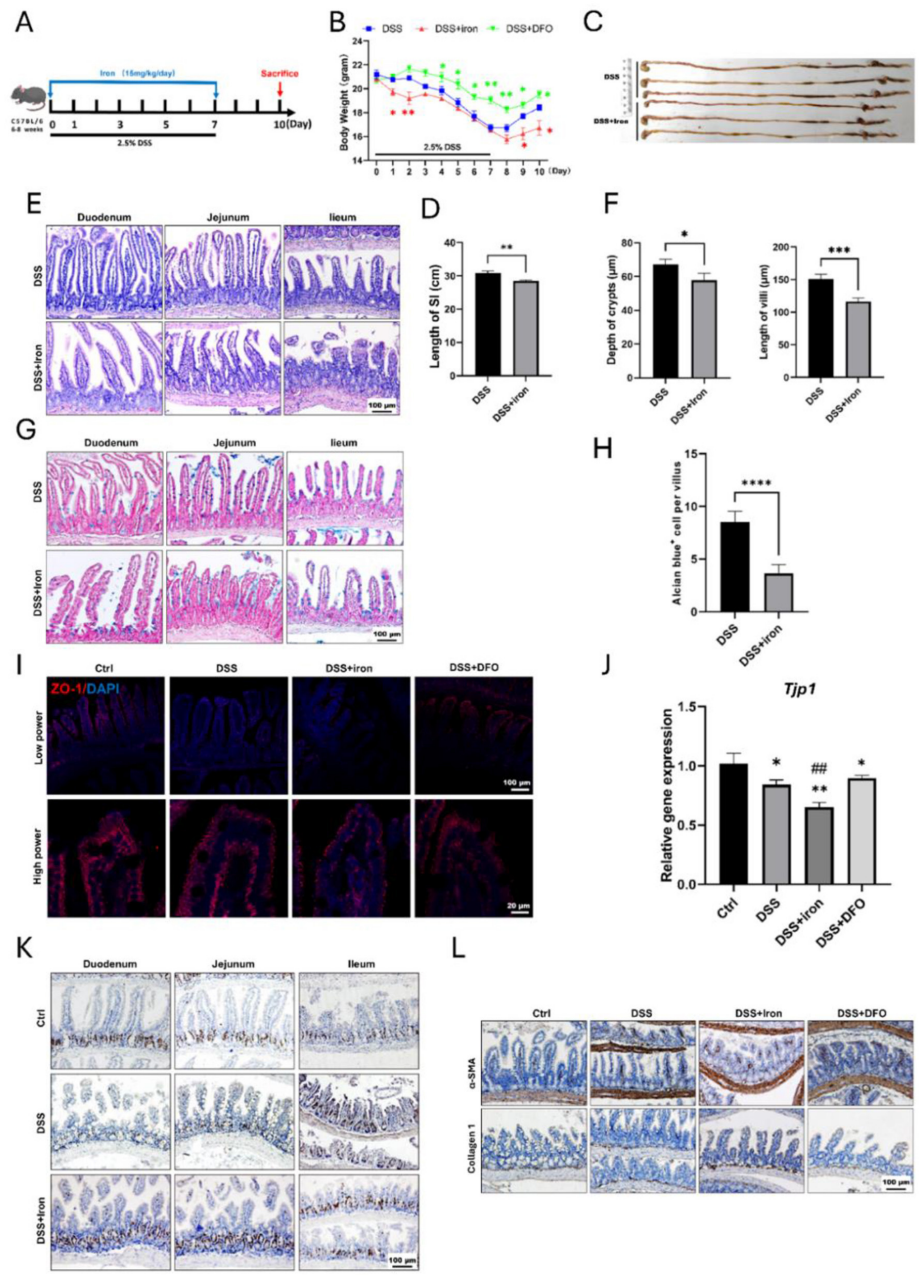
Supplementation of iron retarded the regeneration of intestinal epithelium and enhanced fibrosis. **(A)** Schematic illustration for the strategy of iron supplementation. **(B)** Loss of weight between these two groups (*n* = 6 each group). **(C)** Representative gross gastrointestinal images of two groups. **(D)** Comparison for the length of small intestines of two groups (*n* = 6 per group). **(E)** H&E images of small intestinal tissues. **(F)** Statistical analysis for the depth of crypt and length of villus between two groups (*n* = 6 per group). **(G)** Representative Alcian blue staining of small intestine. **(H)** Quantification of goblet cells within ileum epithelium (*n* = 6 each group). **(I)** IF staining images of ZO-1 within intestinal epithelium staining. **(J)** qPCR results of *TJP1* mRNA expression. **(K)** Representative IHC images of BrdU in different groups. **(L)** Representative IHC images of α-SMA and Collagen 1 in the different groups. *P < 0.05, **P < 0.01, ***P < 0.001, compared to control group or blank group. ****P < 0.0001, compared to control group. ^*##*^*P* < 0.01, compared to DSS group.

Since the previous results showed iron would aggravate intestinal injury, we hypothesized that iron overload was possible to impact the epithelial regeneration. Therefore, we treated mice with DSS and iron for 7 consecutive days and then collected intestines for analysis on day 10 (Figure 3A). It was found that the body weight of iron overload group was recovered more slowly than those DSS-treated mice (Figure 3B). We compared the gross abdominal appearance and found iron-treated mice had slenderer small intestine and thinner intestinal walls (Supplementary Figure S2A). We further isolated the whole gastrointestinal tract, and it was found that the small intestines in iron treated mice were significantly shorter than those of DSS-treated mice (Figures 3C, D). H&E staining demonstrated iron overload caused a poor recovery of intestinal epithelium, which had shorter crypts and villi (Figures 3E, F). In addition, the number of Alcian blue positive goblet cells was also decreased in iron-treated mice (Figures 3G, H), and iron overload also led to fewer staining ZO-1 and less mRNA expression of Tjp1 (Figures 3I, J). Similarly, there were fewer BrdU and olfm4 positive proliferative cells within iron-treated crypts (Figure 3K). Finally, we found that iron overload promoted the expression of a-SMA and Collagen I within lamina propria of ileum (Figure 3L), and it also increased the collagen signals in Masson's staining (Supplementary Figure S2B). Therefore, these results revealed that iron overload could postpone the recovery of DSS-induced intestinal injury and promote intestinal fibrosis.

### 3.4 Iron metabolism directly affected ROS level and epithelial integrity of IEC-6 cells

**Figure 4:**
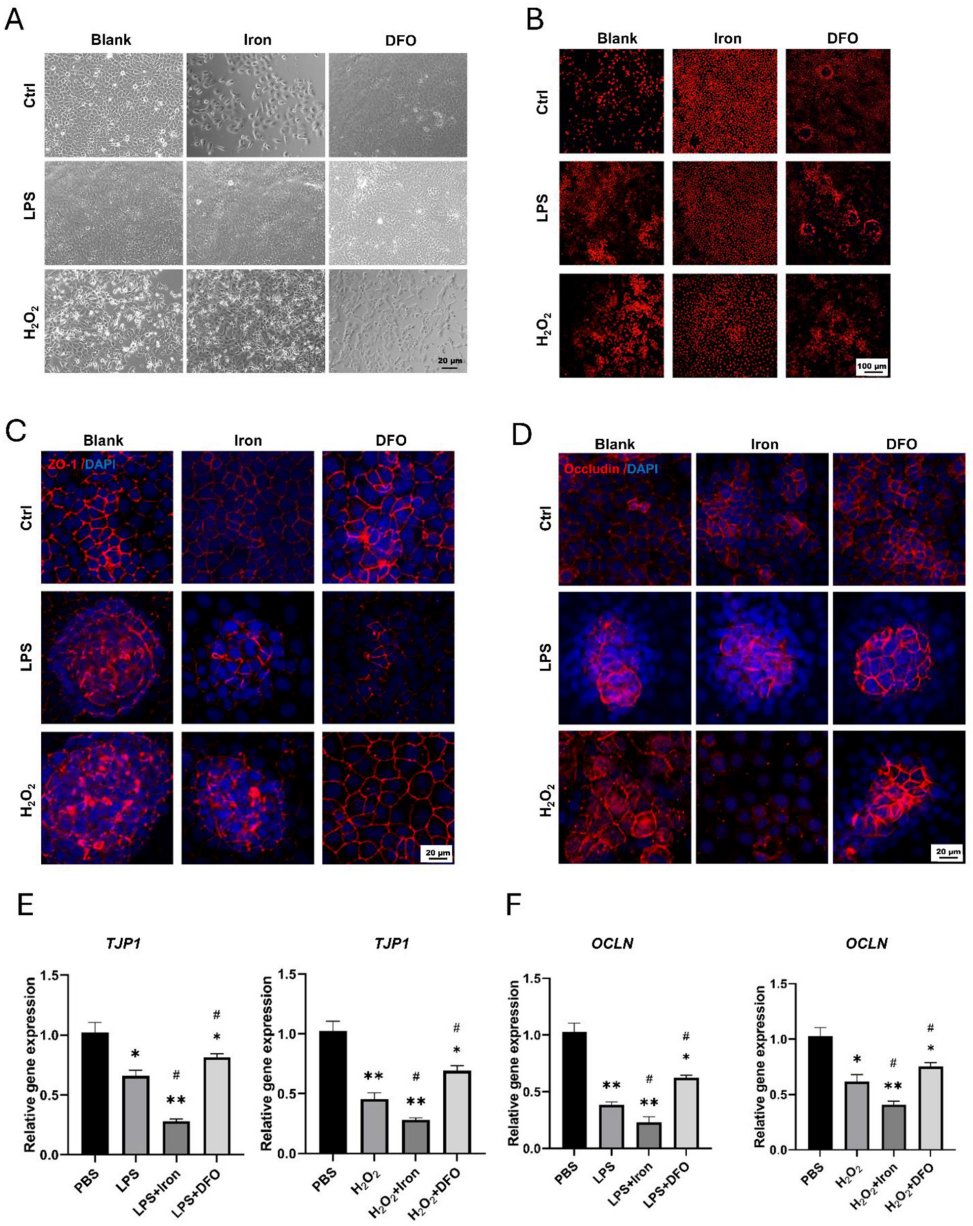
Iron significantly promoted ROS generation and disrupted epithelial integrity of IEC-6 cells. **(A)** Bright field observation of morphology of IEC-6 cells treated by iron or DFO in the presence of LPS or H_2_O_2_. **(B)** DHE staining demonstrated ROS content within IEC-6 cells treated by iron or DFO in different groups. **(C)** Iron and DFO oppositely modulated the staining intensity of ZO-1 in IEC-6 cells treated by LPS or H_2_O_2_. **(D)** Iron and DFO differentially regulated the intensity of Occludin in IEC-6 after LPS or H_2_O_2_ treatment. **(E)** Relative gene expression of *TJP-1* after LPS or H_2_O_2_ treatment in different groups. **(F)** Relative mRNA expression levels of *OCLN* in each group. *P < 0.05, **P < 0.01, compared to PBS group. ^#^*P* < 0.05, compared to LPS or H_2_O_2_ group.

Because there existed many immune cells in intestinal tissues, we wonder whether iron could directly influence epithelial cells using IEC-6, which was epithelial cell line derived from rat jejunal crypt. We observed that both LPS and H_2_O_2_ interrupted the morphology of IEC-6, and 10mM iron promoted such interruption. However, iron chelation by DFO alleviated the damage (Figure 4A). Moreover, it was found that iron treatment apparently increased the ROS level stained by DHE in LPS and H_2_O_2−_treated IEC-6, and DFO decreased the ROS content (Figure 4B). In addition, monolayer IEC-6 could represent the intestinal epithelium. We stained ZO-1 and Occludin to examine how iron modulated their integrity. Interestingly, it was shown that iron overload exacerbated the discontinuous expression of ZO-1 and Occludin caused by LPS and H_2_O_2_. Oppositely, iron chelation by DFO elevated the expression of ZO-1 and Occludin in both immunofluorescent staining and mRNA level (Figures 4C–F). These results proved that iron metabolism was important to prevent ROS-induced epithelial injury and hence keep the integrity of epithelial barrier.

### 3.5 Iron content impaired the formation and growth of intestinal organoids

**Figure 5:**
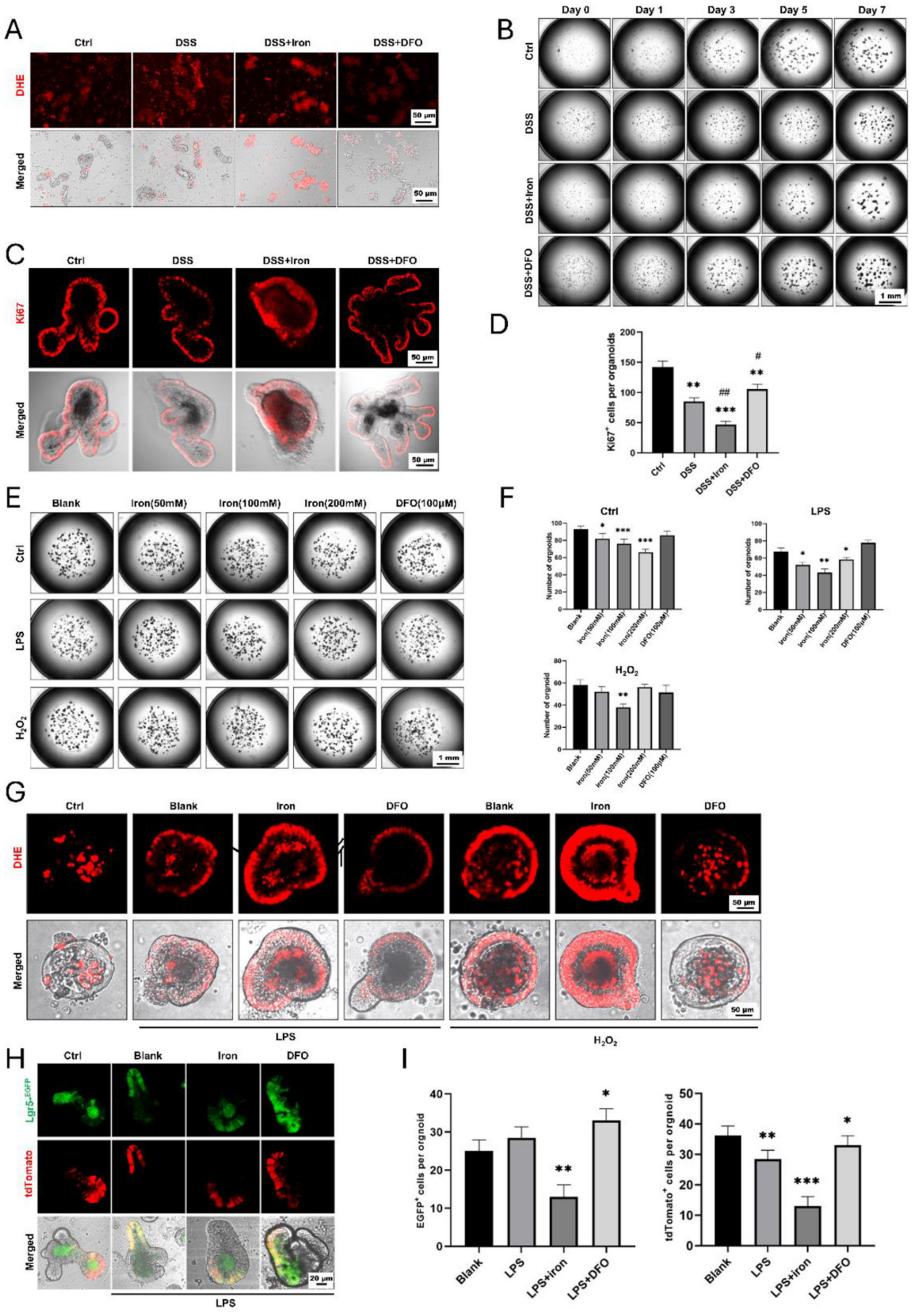
Formation and growth of enteroids from isolated small intestinal crypts in different conditions. **(A)** DHE staining showed different levels of ROS content within freshly isolated crypts. **(B)** Representative growing images of cultured enteroids from small intestine, respectively, treated by DSS, iron, or DFO. **(C)** *In situ* IF staining for Ki67^+^ proliferative cells in enteroids. **(D)** Statistical analysis for the number of Ki67^+^ cells per organoid. **(E)** Images of enteroids treated with iron and DFO after the administration of LPS or H_2_O_2_. **(F)** Quantitative analysis for the number of enteroids in normal medium, LPS medium, and H_2_O_2_ medium treated by iron or DFO. **(G)** DHE staining revealed that iron further increased ROS content in LPS or H_2_O_2_-treated enteroids, and DFO alleviated the level of ROS. **(H)** Lineage tracing of EGFP positive Lgr5^+^ ISCs and their tdTomato^+^ progenies in different groups. **(I)** Statistical analysis of the EGFP positive Lgr5^+^ ISCs and tdTomato^+^ cells. ^*^P < 0.05, ^**^P < 0.01, ^***^P < 0.001, compared to control group or blank group. ^#^*P* < 0.05, ^*##*^*P* < 0.01, compared to DSS group.

Due to the above results, we further investigated the effects of iron metabolism on ISCs using cultured organoids. When we isolated crypts from freshly dissected small intestine, it was found that the ROS content was increased in DSS-treated mice, and iron and DFO oppositely affected the increasing of ROS (Figure 5A). After 7-day culture, fewer enteroids with poor quality were observed in DSS-treated crypts as compared to the healthy enteroids, iron-treated enteroids even grew worse, but DFO-treated crypts showed better growth (Figure 5B, Supplementary Figure S3A). Next, we stained Ki67 to evaluate the proliferation and found an obvious decreasing in DSS-treated enteroids. Interestingly, iron-treated enteroids had less Ki67^+^ cells, and DFO group had more Ki67^+^ cells (Figures 5C, D). In addition, we also treated enteroids grown for 3 days with iron and DFO in combination with LPS or H_2_O_2_. It was discovered that iron caused a dose-dependent decrease of organoid number in control group and LPS- or H_2_O_2_-treated enteroids, but DFO preserved the quantity of enteroids (Figures 5E, F). Subsequently, we investigated the ROS level in enteroids by DHE staining. The results showed relatively low ROS signals in healthy enteroids, and LPS and H_2_O_2_ increased ROS level. Meanwhile, iron promoted the increase of ROS, but DFO inhibited (Figure 5G). Finally, we used Lgr5-tdtomato to directly reflect the influence of iron content on the activity of ISCs. It was shown that during LPS or H_2_O_2_ treatment, iron further inhibited the differentiation of EGFP positive Lgr5^+^ ISCs and DFO alleviated such an inhibition (Figures 5H, I, Supplementary Figure S3B). Therefore, the data suggested that iron content was important to maintain activity of ISCs both *in vivo* and *in vitro* to support the formation and growth of enteroids during inflammatory stimulation.

### 3.6 Iron impacted the activation of STAT3 and ERK signaling pathways and immune cell infiltration within small intestine

**Figure 6:**
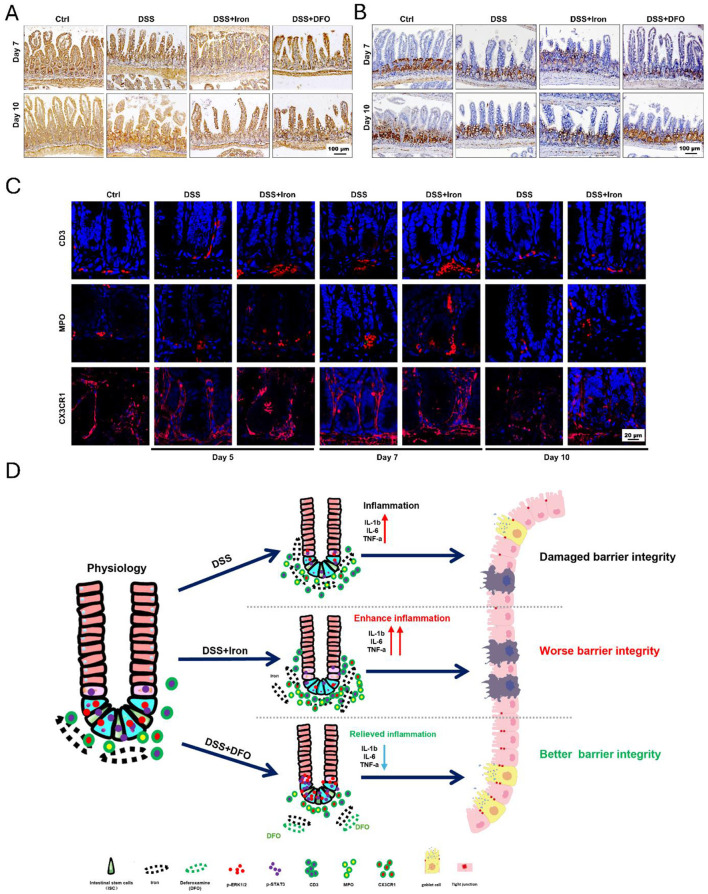
Iron impaired the activity of STAT3 and ERK signaling pathway and the infiltration of immune cells during DSS-induced colitis. **(A)** Representative images of IHC staining against p-STAT3 in small intestine at day 7 and day 10 after DSS treatment. **(B)** IHC images of p-ERK1/2 at day 7 and day 10 in each group during experimental colitis (Bar = 100 μm). **(C)** Immunofluorescent (IF) staining of CD3, MPO, and CX3CR1 in DSS-treated small intestine with or without iron supplementation at different time points. CD3: T cells, MPO: neutrophils, CX3CR1: macrophages (Bar = 20 μm). **(D)** Proposed graphic model of how iron modulated barrier integrity and the function of ISCs in small intestine during DSS-induced experimental colitis.

To determine the mechanisms why iron metabolism would affect small intestinal injury during DSS-induced colitis, we further examined the signaling transduction in epithelial cells and the infiltration of immune cells. It has been known that STAT3 and ERK1/2 are two critical pathways of intestinal epithelium due to their modulation on signaling transduction and proliferation activity of ISCs (37). We performed IHC staining against p-STAT3 and p-ERK1/2 to examine how the activity of STAT3 and ERK1/2 pathways changed during intestinal inflammation. Interestingly, it was revealed that DSS treatment caused an increased expression of p-STAT3 within epithelium and iron promoted such an increase at day 7, but iron chelation by DFO restricted the hyperactivation of STAT3 pathway. When DSS was removed at day 10, there was still a high staining of p-STAT3, and DFO group had almost normal level of p-STAT3 (Figure 6A). ERK signaling was mainly detected in crypts, whose expression was seriously inhibited by DSS-induced inflammation, and high level of iron content exacerbated such inhibition at day 7. Oppositely, DFO treatment partially saved p-ERK1/2 expression and maintained the histology of intestinal epithelium at day 7. After withdrawal of DSS, iron-treated intestine had a retarded recovery of p-ERK1/2 compare with DSS group, but DFO group showed even a higher recovery for the expression of p-ERK1/2 (Figure 6B). It has been reported that iron accumulation could promote the generation of ROS in immune cells, such as macrophages. Then, we stained the infiltration of inflammatory cells and observed that the supplementation of iron greatly promoted the infiltration of immune cells into small intestine, including CD3^+^ T cells and MPO^+^ neutrophils. These infiltrated immune cells might enhance local inflammatory response and thus promoted intestinal injury. In addition, CX3CR1^+^ macrophages are considered as resident macrophages, and we found these spindle-like macrophages became short, indicating their polarization might be changed due to iron treatment (Figure 6C). Therefore, these data supported that iron could significantly interrupt STAT3 and ERK signaling pathway and strengthen inflammation-induced damages to epithelial integrity of small intestine (Figure 6D).

## 4 Discussion

It has been reported that the morbidity of IBD is increasing due to dietary pattern and lifestyle with social development, so UC, as the majority of IBD, is attracting more scientific attention during the past decades. UC brings the patients many inconveniences, and it costs more and more healthcare resources (38). It is of great value to reveal the exact pathological mechanisms and develop the effective therapeutic methods for UC (39, 40). However, almost all the existed reports have been focused on the colonic injury, and little attention has been paid to the changes within small intestine, which is the main organ responsible for nutrient absorption, host immunity, and mucosal barrier.

Iron deficiency anemia (IDA) is often present in patients with IBD, and the main source of excess iron loss is intestinal bleeding (26), reduced iron absorption (2), drugs used for IBD such as methotrexate, thiopurine, sulfasalazine, and thiopurine proton pump inhibitors (41–43), and vitamin deficiencies such as B12, folate, and vitamin D (42, 44). Correcting the underlying cause and supplementing adequate iron stores is required for IDA and IBD treatment, and the purpose is to completely normalize anemia and iron stores (2). Iron is mainly ingested through the diet, which is directly involved in regulating the composition and metabolic activities of the gut microbiota and is related to the microbial regulation of the immune system (45). The proliferation and growth of almost all microbiota, including fungi and pathogenic species, is dependent on the utilization of unabsorbed dietary siron. In addition, iron deficiency is associated with reduced abundance and diversity of gut microbiota (46). Oral iron is the most commonly used iron replacement therapy due to its low cost, availability, and ease of administration (47). In summary, diet, microbes, and iron metabolism present complex interactions in the gut, further revealing the complexity of iron metabolism.

Several studies have discussed some changes of small intestine during colitis. For example, Yazbeck et al. showed inflammatory damage would extend to small intestine (20). BR11, which was a microbial fermented product, increased epithelial proliferation (21). Schmitt et al. reported that DSS-induced colitis even triggered the dedifferentiation of terminally differentiated Paneth cells to reconstitute the damaged epithelium of small intestine (22). However, we believe that it is critical to understand the basic pathologic process of small intestinal injury to propose any useful and specific interventions. Therefore, in this study, we characterized the whole process of small intestinal injury during DSS-induced experimental colitis.

We utilized drinking of DSS to establish experimental colitis. DSS-induced colitis depends on the molecular weight and concentration of DSS and the period of treatment (48). Here, we treated mice with 2.5% DSS (molecular weight: 36,000–50,000) for 7 days, which is the most frequently regimen in the previous study. Interestingly, our results showed that the length of small intestine in mice was significantly reduced with the drinking of DSS (Figure 1D). Histological staining also confirmed the injury of small intestinal epithelium, such as shrunk villi, atrophic crypts, less mucus-secreting goblet cells, and downregulated expression of tight junction markers ZO-1 and Occludin (Figures 1G–L). Meanwhile, the degree of DSS-induced injury in small intestine varied depending on different segments, and distal ileum showed the most serious damage. Therefore, we concluded that small intestine was impaired during DSS-induced colitis.

Iron is an essential trace element for both health and the pathology of diseases in mammals (49). It is inevitable to a wide variety of biological processes. For example, oxygen transport in red blood cells depends on the iron-rich hemoglobin, and ATP generation in mitochondria also needs the involvement of iron. However, inappropriate iron supplementation may result in harm to tissue homeostasis and regeneration due to the over production of ROS and ferroptosis. Although many IBD patients always have a deficiency of iron caused by chronic bleeding and insufficient absorption, overload of iron has been identified to exacerbate intestinal injury (50). Recently, using DSS-induced IBD model of drosophila, Usman et al. demonstrated that ferrous sulfate increased lipid peroxidation and bathophenanthroline disulfonate (BPS), which was also an iron chelator, protected flies from DSS-induced IBD (51). In the current study, we found overload of iron was harmful to small intestine, which worsened the epithelial injury during DSS treatment (Figure 2) and delayed the epithelial regeneration after withdrawal of DSS (Figure 3). Meanwhile, to rule out the non-specific effects of iron and DFO, we used a control group without DSS treatment and found the current strategy of iron supplementation or depletion would not impair the healthy small intestine (Supplementary Figure S4). Although iron supplementation alleviated DSS-induced decreasing of RBC count (Supplementary Figure S5A), it increased the intestinal permeability as shown by FITC-dextran (FD4) assay (Supplementary Figure S5B). Iron also enhanced the cytotoxicity of LPS-treated IEC-6 cells and enteroids, which were determined by LDH releasing assay (Supplementary Figures S5C, D). These results suggest that although most patients with IBD require iron supplementation to improve anemia, iron overload would damage small intestine. Therefore, the strategy of iron supplementation in clinical practice needs to be more cautious and consider more factors.

ISCs are the hub of epithelial renewal and regeneration. Dysfunction of ISCs is tightly related to less generation of new epithelial cells, which restricts the regeneration speed of intestinal barrier (52, 53). For example, ionizing radiation caused rapid apoptosis of ISCs, and we have shown that berberine (BBR) or intravenous immunoglobulin (IVIg) could boost the activity of ISCs to promote epithelial regeneration (37, 54). In the circumstance of DSS-induced colitis, we found iron supplementation resulted in more infiltration of inflammatory cells (e.g., CD3^+^ T cells and MPO^+^ neutrophils), and it also changed the morphology of CX3CR1^+^ resident macrophages surrounding intestinal crypts (Figure 6C). Over-dosing iron could directly impair the biological activity of intestinal epithelial cells. Using IEC-6, we showed iron modulation would affect the proliferation, ROS content, and tight junction (Figure 4). Moreover, it was demonstrated that isolated crypts from mice with DSS-induced colitis formed less enteroids compared to healthy crypts, and iron-treated enteroids even grew worse (Figure 5). Importantly, it was demonstrated that iron overload was able to inhibit the differentiation of EGFP positive Lgr5^+^ ISCs (Figures 5H, I, Supplementary Figure S3B). Therefore, these data supported that iron content would greatly influence barrier integrity and ISC function via both enhancing inflammation and direct effects.

There are several shortcomings in the current study. First of all, we used the doses of iron supplementation and DFO following the previous study (35). Given iron can exhibit both beneficial and detrimental effects *in vivo*, iron doses should be optimized before the study. Second, the current study mainly focused on the acute settings of DSS-induced colitis. However, many clinical IBD patients suffer from a chronic disease process, so a longitudinal analysis of iron regulation (both supplementation and depletion) is worth of great value for more exact determination of whether these effects could persist or lead to adaptive compensatory. Third, gut microbiota would not only impair the absorption and transportation of iron, but their diversity is also affected by the iron supplementation or depletion. A sequencing analysis of microbiome shift due to the iron regulation would contribute to further understanding of the observed results. Finally, there is lack of monitoring the concentration of iron in the blood, which will be able to directly reflect the bioavailability of iron. The function of hematopoiesis should also be considered during the administration of iron supplementation and DFO. It would be more helpful if we can collect some clinical biopsy samples to validate the findings.

## 5 Conclusion

In summary, our study revealed that small intestine was also significantly impaired during the process of experimental colitis, and iron overload was negative to intestinal epithelial barrier and ISCs. This study emphasizes that it is of great importance to investigate cellular and molecular changes in small intestine during the process of experimental colitis, and appropriate and careful iron uptake is vital to control DSS-induced intestinal injury. The strategy of iron supplementation in clinical practice needs to be more cautious and consider more factors.

## Data Availability

The original contributions presented in the study are included in the article/Supplementary material, further inquiries can be directed to the corresponding authors.

## References

[1] M'koma AE. Inflammatory bowel disease: an expanding global health problem. Clin Med Insights Gastroenterol. (2013) 6:33–47. 10.4137/CGast.S12731 24833941 PMC4020403

[2] Jimenez KM Gasche C. Management of Iron Deficiency Anaemia in Inflammatory Bowel Disease. Acta Haematol. (2019) 142:30–6. 10.1159/000496728 30970351

[3] D'amico F Peyrin-Biroulet L Danese S. Oral Iron for IBD Patients: Lessons Learned at Time of COVID-19 Pandemic. J Clin Med. (2020) 9:1536. 10.3390/jcm9051536 32438763 PMC7290728

[4] De Silva AD Mylonaki M Rampton DS. Oral iron therapy in inflammatory bowel disease: usage, tolerance, and efficacy. Inflamm Bowel Dis. (2003) 9:316–20. 10.1097/00054725-200309000-00005 14555915

[5] Gomollon F Gisbert JP. Anemia and inflammatory bowel diseases. World J Gastroenterol. (2009) 15:4659–65. 10.3748/wjg.15.4659 19787829 PMC2754514

[6] Hindryckx P Amininejad L Van De Vijver E Bossuyt P Belgian Group for IBD Research and Development. Belgian recommendations for the management of anemia in patients with inflammatory bowel disease. Acta Gastroenterol Belg. (2014) 77:333–44. 25509205

[7] Itzkowitz SH Yio X. Inflammation and cancer IV. Colorectal cancer in inflammatory bowel disease: the role of inflammation. Am J Physiol Gastrointest Liver Physiol. (2004) 287:G7–17. 10.1152/ajpgi.00079.2004 15194558

[8] Jia Q Lupton JR Smith R Weeks BR Callaway E Davidson LA. Reduced colitis-associated colon cancer in Fat-1 (n-3 fatty acid desaturase) transgenic mice. Cancer Res. (2008) 68:3985–91. 10.1158/0008-5472.CAN-07-6251 18483285 PMC2648804

[9] Rosen JM Jordan CT. The increasing complexity of the cancer stem cell paradigm. Science. (2009) 324:1670–3. 10.1126/science.1171837 19556499 PMC2873047

[10] Voelker R. What is ulcerative colitis? JAMA. (2024) 331:716. 10.1001/jama.2023.23814 38306113

[11] Menconi A Hernandez-Velasco X Vicuna EA Kuttappan VA Faulkner OB Tellez G. Histopathological and morphometric changes induced by a dextran sodium sulfate (DSS) model in broilers. Poult Sci. (2015) 94:906–11. 10.3382/ps/pev054 25743415

[12] Ginzel M Feng X Kuebler JF Klemann C Yu Y Von Wasielewski R. Dextran sodium sulfate (DSS) induces necrotizing enterocolitis-like lesions in neonatal mice. PLoS One. (2017) 12:e0182732. 10.1371/journal.pone.0182732 28817583 PMC5560643

[13] Ohkusa T. Production of experimental ulcerative colitis in hamsters by dextran sulfate sodium and changes in intestinal microflora. Nihon Shokakibyo Gakkai Zasshi. (1985) 82:1327–36. 2411981

[14] Okayasu I Hatakeyama S Yamada M Ohkusa T Inagaki Y Nakaya R. A novel method in the induction of reliable experimental acute and chronic ulcerative colitis in mice. Gastroenterology. (1990) 98:694–702. 10.1016/0016-5085(90)90290-H 1688816

[15] Geier GR. Revolution? Minn Med. (2005) 88:17. 16022401

[16] Parang B Barrett CW Williams CS. AOM/DSS model of colitis-associated cancer. Methods Mol Biol. (2016) 1422:297–307. 10.1007/978-1-4939-3603-8_26 27246042 PMC5035391

[17] Xu J Zhou L Ji L Chen F Fortmann K Zhang K. The REGgamma-proteasome forms a regulatory circuit with IkappaBvarepsilon and NFkappaB in experimental colitis. Nat Commun. (2016) 7:10761. 10.1038/ncomms10761 26899380 PMC4764899

[18] Elsheikh W Flannigan KL Mcknight W Ferraz JG Wallace JL. Dextran sulfate sodium induces pan-gastroenteritis in rodents: implications for studies of colitis. J Physiol Pharmacol. (2012) 63:463–9. 23211300

[19] Omidi-Ardali H Lorigooini Z Soltani A Balali-Dehkordi S Amini-Khoei H. Inflammatory responses bridge comorbid cardiac disorder in experimental model of IBD induced by DSS: protective effect of the trigonelline. Inflammopharmacology. (2019) 27:1265–73. 10.1007/s10787-019-00581-w 30924005

[20] Yazbeck R Howarth GS Butler RN Geier MS Abbott CA. Biochemical and histological changes in the small intestine of mice with dextran sulfate sodium colitis. J Cell Physiol. (2011) 226:3219–24. 10.1002/jcp.22682 21351101

[21] Geier MS Smith CL Butler RN Howarth GS. Small-intestinal manifestations of dextran sulfate sodium consumption in rats and assessment of the effects of Lactobacillus fermentum BR11. Dig Dis Sci. (2009) 54:1222–8. 10.1007/s10620-008-0495-4 19005763

[22] Schmitt M Schewe M Sacchetti A Feijtel D Van De Geer WS Teeuwssen M. Paneth cells respond to inflammation and contribute to tissue regeneration by acquiring stem-like features through SCF/c-kit signaling. Cell Rep. (2018) 24:2312–28.e7. 10.1016/j.celrep.2018.07.085 30157426

[23] Cerantola S Faggin S Caputi V Bosi A Banfi D Rambaldo A. Small intestine neuromuscular dysfunction in a mouse model of dextran sulfate sodium-induced ileitis: Involvement of dopaminergic neurotransmission. Life Sci. (2022) 301:120562. 10.1016/j.lfs.2022.120562 35487304

[24] Pai MH Liu JJ Yeh SL Chen WJ Yeh CL. Glutamine modulates acute dextran sulphate sodium-induced changes in small-intestinal intraepithelial gammadelta-T-lymphocyte expression in mice. Br J Nutr. (2014) 111:1032–9. 10.1017/S0007114513003425 24229607

[25] Kong X Wang X Qin Y Han J. Effects of sunset yellow on proliferation and differentiation of intestinal epithelial cells in murine intestinal organoids. J Appl Toxicol. (2021) 41:953–63. 10.1002/jat.4080 33063357

[26] Gasche C Lomer MC Cavill I Weiss G. Iron, anaemia, and inflammatory bowel diseases. Gut. (2004) 53:1190–7. 10.1136/gut.2003.035758 15247190 PMC1774131

[27] Walter S Mertens C Muckenthaler MU Ott C. Cardiac iron metabolism during aging - Role of inflammation and proteolysis. Mech Ageing Dev. (2023) 215:111869. 10.1016/j.mad.2023.111869 37678569

[28] Velasquez J Wray AA. Deferoxamine StatPearls. Treasure Island (FL): StatPearls. (2024).32491586

[29] Nairz M Weiss G. Iron in infection and immunity. Mol Aspects Med. (2020) 75:100864. 10.1016/j.mam.2020.100864 32461004

[30] Kernan KF Carcillo JA. Hyperferritinemia and inflammation. Int Immunol. (2017) 29:401–9. 10.1093/intimm/dxx031 28541437 PMC5890889

[31] An HS Yoo JW Jeong JH Heo M Hwang SH Jang HM. Lipocalin-2 promotes acute lung inflammation and oxidative stress by enhancing macrophage iron accumulation. Int J Biol Sci. (2023) 19:1163–77. 10.7150/ijbs.79915 36923935 PMC10008694

[32] Yatmark P Morales NP Chaisri U Wichaiyo S Hemstapat W Srichairatanakool S. Effects of iron chelators on pulmonary iron overload and oxidative stress in beta-thalassemic mice. Pharmacology. (2015) 96:192–9. 10.1159/000438994 26316149

[33] Cheng H Feng D Li X Gao L Tang S Liu W. Iron deposition-induced ferroptosis in alveolar type II cells promotes the development of pulmonary fibrosis. Biochim Biophys Acta Mol Basis Dis. (2021) 1867:166204. 10.1016/j.bbadis.2021.166204 34175430

[34] Meng X Huang W Mo W Shu T Yang H Ning H. ADAMTS-13-regulated nuclear factor E2-related factor 2 signaling inhibits ferroptosis to ameliorate cisplatin-induced acute kidney injuy. Bioengineered. (2021) 12:11610–21. 10.1080/21655979.2021.1994707 34666603 PMC8810018

[35] Shubin Wang XL Lu X Jinyi L Dengqun L. Phase-dependent iron depletion differentially regulates the niche of intestinal stem cells in experimental colitis via ERK/STAT3 signaling pathway Front Immunol. (2025) 16:1537651. 10.3389/fimmu.2025.1537651 39949764 PMC11822217

[36] Chai S Liu K Feng W Liu T Wang Q Zhou R. Activation of G protein-coupled estrogen receptor protects intestine from ischemia/reperfusion injury in mice by protecting the crypt cell proliferation. Clin Sci. (2019) 133:449–64. 10.1042/CS20180919 30705108

[37] Tu S Huang Y Tian H Xu L Wang X Huang L. Berberine enhances the function of intestinal stem cells in healthy and radiation-injured mice. Int Immunopharmacol. (2024) 136:112278. 10.1016/j.intimp.2024.112278 38815353

[38] Kiesslich R Duckworth CA Moussata D Gloeckner A Lim LG Goetz M. Local barrier dysfunction identified by confocal laser endomicroscopy predicts relapse in inflammatory bowel disease. Gut. (2012) 61:1146–53. 10.1136/gutjnl-2011-300695 22115910 PMC3388727

[39] Capaldo CT Powell DN Kalman D. Layered defense: how mucus and tight junctions seal the intestinal barrier. J Mol Med (Berl). (2017) 95:927–34. 10.1007/s00109-017-1557-x 28707083 PMC5548832

[40] Ramos GP Papadakis KA. Mechanisms of disease: inflammatory bowel diseases. Mayo Clin Proc. (2019) 94:155–65. 10.1016/j.mayocp.2018.09.013 30611442 PMC6386158

[41] Ganz T Nemeth E. Hepcidin and iron homeostasis. Biochim Biophys Acta. (2012) 1823:1434–43. 10.1016/j.bbamcr.2012.01.014 22306005 PMC4048856

[42] Nielsen OH Ainsworth M Coskun M Weiss G. Management of iron-deficiency anemia in inflammatory bowel disease: a systematic review. Medicine (Baltimore). (2015) 94:e963. 10.1097/MD.0000000000000963 26061331 PMC4616486

[43] Niepel D Klag T Malek NP Wehkamp J. Practical guidance for the management of iron deficiency in patients with inflammatory bowel disease. Therap Adv Gastroenterol. (2018) 11:1756284818769074. 10.1177/1756284818769074 29760784 PMC5946590

[44] Gisbert JP Gomollon F. Common misconceptions in the diagnosis and management of anemia in inflammatory bowel disease. Am J Gastroenterol. (2008) 103:1299–307. 10.1111/j.1572-0241.2008.01846.x 18477354

[45] Belkaid Y Hand TW. Role of the microbiota in immunity and inflammation. Cell. (2014) 157:121–41. 10.1016/j.cell.2014.03.011 24679531 PMC4056765

[46] Mayneris-Perxachs J Moreno-Navarrete JM Fernandez-Real JM. The role of iron in host-microbiota crosstalk and its effects on systemic glucose metabolism. Nat Rev Endocrinol. (2022) 18:683–98. 10.1038/s41574-022-00721-3 35986176

[47] Gomez-Ramirez S Brilli E Tarantino G Munoz M. Sucrosomial((R)) iron: a new generation iron for improving oral supplementation. Pharmaceuticals. (2018) 11. 10.3390/ph11040097 30287781 PMC6316120

[48] Egger B Bajaj-Elliott M Macdonald TT Inglin R Eysselein VE Buchler MW. Characterisation of acute murine dextran sodium sulphate colitis: cytokine profile and dose dependency. Digestion. (2000) 62:240–8. 10.1159/000007822 11070407

[49] Huo C Li G Hu Y Sun H. The impacts of iron overload and ferroptosis on intestinal mucosal homeostasis and inflammation. Int J Mol Sci. (2022) 23:14195. 10.3390/ijms232214195 36430673 PMC9697168

[50] Gu K Wu A Yu B Zhang T Lai X Chen J. Iron overload induces colitis by modulating ferroptosis and interfering gut microbiota in mice. Sci Total Environ. (2023) 905:167043. 10.1016/j.scitotenv.2023.167043 37717771

[51] Usman D Abubakar MB Ibrahim KG Imam MU. Iron chelation and supplementation: A comparison in the management of inflammatory bowel disease using drosophila. Life Sci. (2024) 336:122328. 10.1016/j.lfs.2023.122328 38061132

[52] Hageman JH Heinz MC Kretzschmar K Van Der Vaart J Clevers H Snippert HJG. Intestinal regeneration: regulation by the microenvironment. Dev Cell. (2020) 54:435–46. 10.1016/j.devcel.2020.07.009 32841594

[53] Lei X Xu Z Huang Y Huang L Lang J Qu M. Regulation of mitochondrial quality control of intestinal stem cells in homeostasis and diseases. Antioxid Redox Signal. (2024) 42:494–511. 10.1089/ars.2023.0489 39225500

[54] He J Jiang P Ma L Liu F Fu P Du X. Intravenous immunoglobulin protects the integrity of the intestinal epithelial barrier and inhibits ferroptosis induced by radiation exposure by activating the mTOR pathway. Int Immunopharmacol. (2024) 131:111908. 10.1016/j.intimp.2024.111908 38518594

