# Supplementary material for: Iron modulates barrier integrity and stem cell function of small intestine during experimental colitis
Source: Front Nutr. 2025 May 9;12:1545956. doi: 10.3389/fnut.2025.1545956 (PMC12100934; doi:10.3389/fnut.2025.1545956)
Supplement: Supplementary file 1 [file Data_Sheet_1.docx]

**Iron modulates barrier integrity and stem cell function of small intestine** **during experimental colitis**

**Shubin Wang^1†^,** **Xiangjie Yang^1†^, Xiangjun Liu^1†^, Qin Wen^1^, Lu Xu^1^, Mei Feng^1,3*^, Jinyi Lang^1^*, Dengqun Liu^1,2^***

^1^ Radiation Oncology Key Laboratory of Sichuan Province, Sichuan Cancer Hospital & Institute, Sichuan Provincial Engineering Research Center for Tumor Organoids and Clinical Transformation, Sichuan Clinical Research Center for Cancer, Sichuan Cancer Center, School of Medicine, University of Electronic Science and Technology of China, Chengdu 610041, China.

^2^ Department of Experimental Research, Sichuan Cancer Hospital & Institute, Sichuan Provincial Engineering Research Center for Tumor Organoids and Clinical Transformation, Sichuan Clinical Research Center for Cancer, Sichuan Cancer Center, School of Medicine, University of Electronic Science and Technology of China, Chengdu 610041, China.

^3^ Department of Medical Oncology, The Third People’s Hospital of Sichuan Province, Chengdu, 610100, China.

† These authors contributed equally to this work.

*** Correspondence:**
Prof. Mei Feng, E-mail: [fengmei1@scszlyy.org.cn](mailto:fengmei1@scszlyy.org.cn);

Prof. Jinyi Lang, E-mail: [langjinyi@scszlyy.org](mailto:langjinyi@scszlyy.org)

Prof. Dengqun Liu, E-mail: [dengqunliu@uestc.edu.cn](mailto:dengqunliu@uestc.edu.cn)

† These authors have contributed equally to this work

**Supplementary materials**

**Table S1. The information of antibodies for immunostaining**

| **Antibody** | **Host** | **Supplier** | **Catalog** |
| --- | --- | --- | --- |
| BrdU | Mouse | Biolegend | 364102 |
| Collagen I | Rabbit | Proteintech | 14695-1-AP |
| p-ERK1/2 | Rabbit | Cell Signaling | 4370 |
| Ki67 | Rabbit | Abcam | ab16667 |
| Olfm4 | Rabbit | Abcam | 39141 |
| Occludin | Rabbit | Proteintech | 13409-1-AP |
| a-SMA | mouse | Boster | BM0002 |
| p-STAT3 | Rabbit | Cell Signaling | 9145 |
| ZO-1 | Rabbit | Proteintech | 13409-1-AP |

**Table S2. Primer sequences used for qPCR assay**

| **Gene** | | **Forward primer** | **Reverse primer** |
| --- | --- | --- | --- |
| IL-1b | ACCTCACAAGCAGAGCACAA | | TTGGCCGAGGACTAAGGAGT |
| IL-10 | TAAGGCTGGCCACACTTGAG | | GTTTTCAGGGATGAAGCGGC |
| Lgr5 | CCTACTCGAAGACTTACCCAGT | | GCATTGGGGTGAATGATAGCA |
| Mki67 | ATCATTGACCGCTCCTTTAGGT | | GCTCGCCTTGATGGTTCCT |
| Ocln | TCCGGCCGCCAAGGTTC | | CATAGCCTCTGTCCCAAGCAA |
| Tjp1 | GCCTTGGCCTAGCATACACA | | GGTAAGGCATTCCTGCTGGT |
| TNF-a | ATGGCCTCCCTCTCATCAGT | | TTTGCTACGACGTGGGCTAC |
| Actb | GTGACGTTGACATCCGTAAAGA | | GCCGGACTCATCGTACTCC |

**Figure legend**

**Fig. S1**


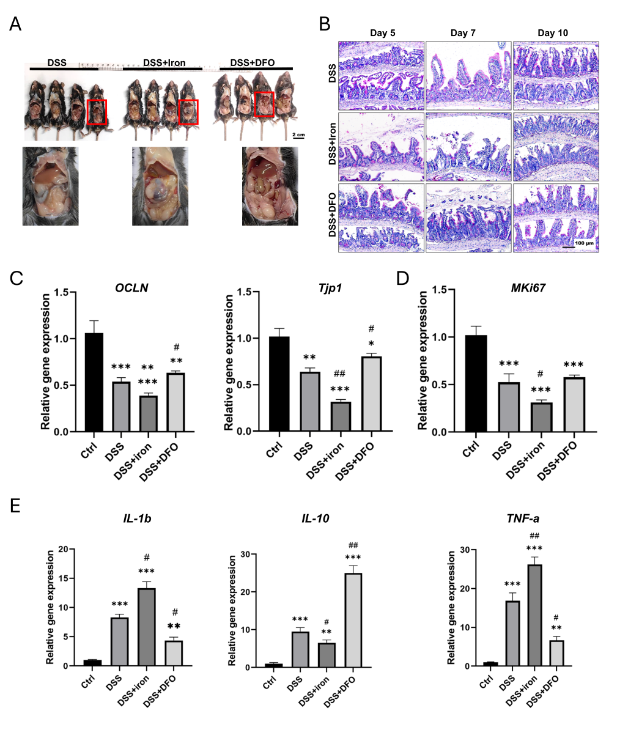


**Fig. S1. Intervention of iron content affected epithelial injury of small intestine during DSS-induced colitis.** (A) Gross appearance of gastrointestinal tract treated for 7 days. (B) PAS staining of small intestine at different periods of colitis. (C) mRNA expression levels of *Tjp1*, *OCLN* in different groups. (D) qPCR examination for *MKi67* in different groups. (E) Relative gene expression levels of *IL-1b*, *TNF-a* and *IL-10* were examined by qPCR assay. **: *P*< 0.01, ***: *P*< 0.001, compared to control group or blank group. #: *P*< 0.05, ##: *P*< 0.01, compared to DSS group.

**Fig. S2**


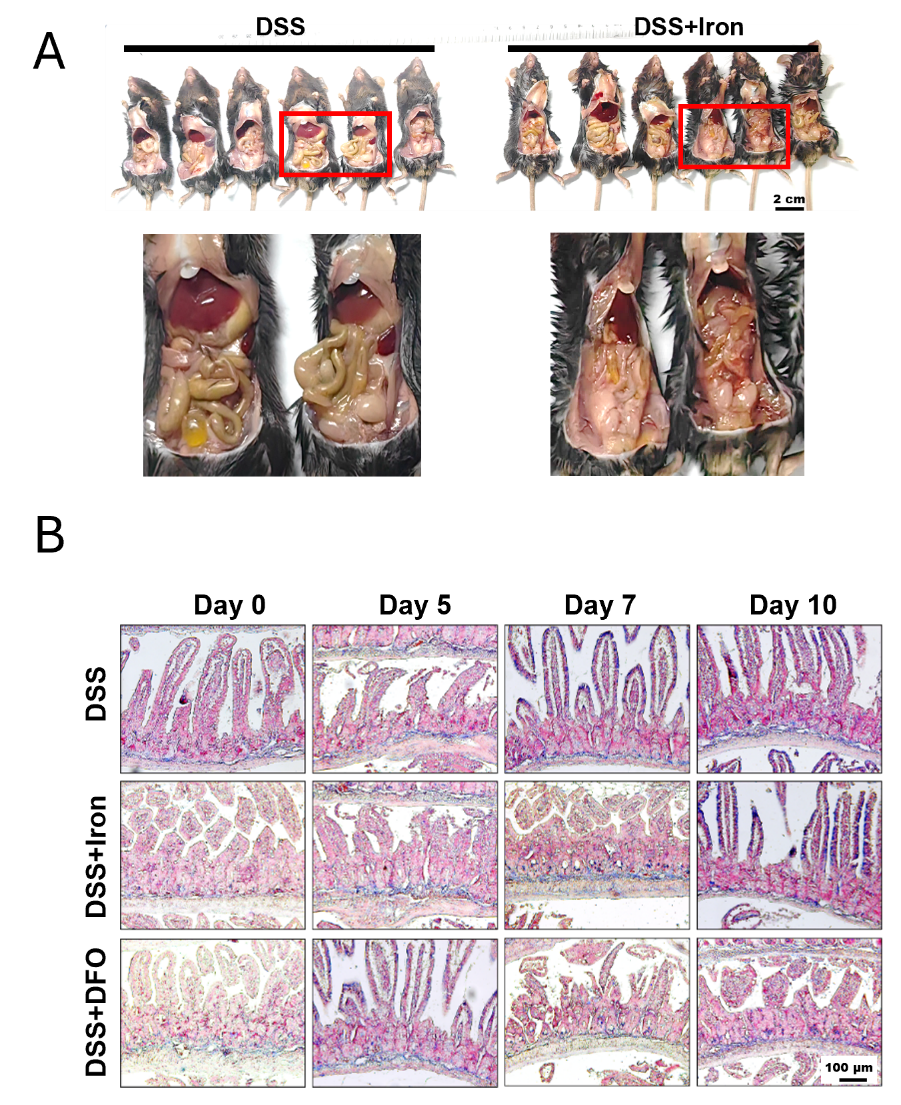


**Fig. S2. Iron overload delayed the regeneration of small intestine and promoted intestinal fibrosis.** (A) Gross images of abdominal cavity between DSS and iron supplementation groups. (B) Masson staining of small intestinal tissues of different groups.

**Fig. S3**


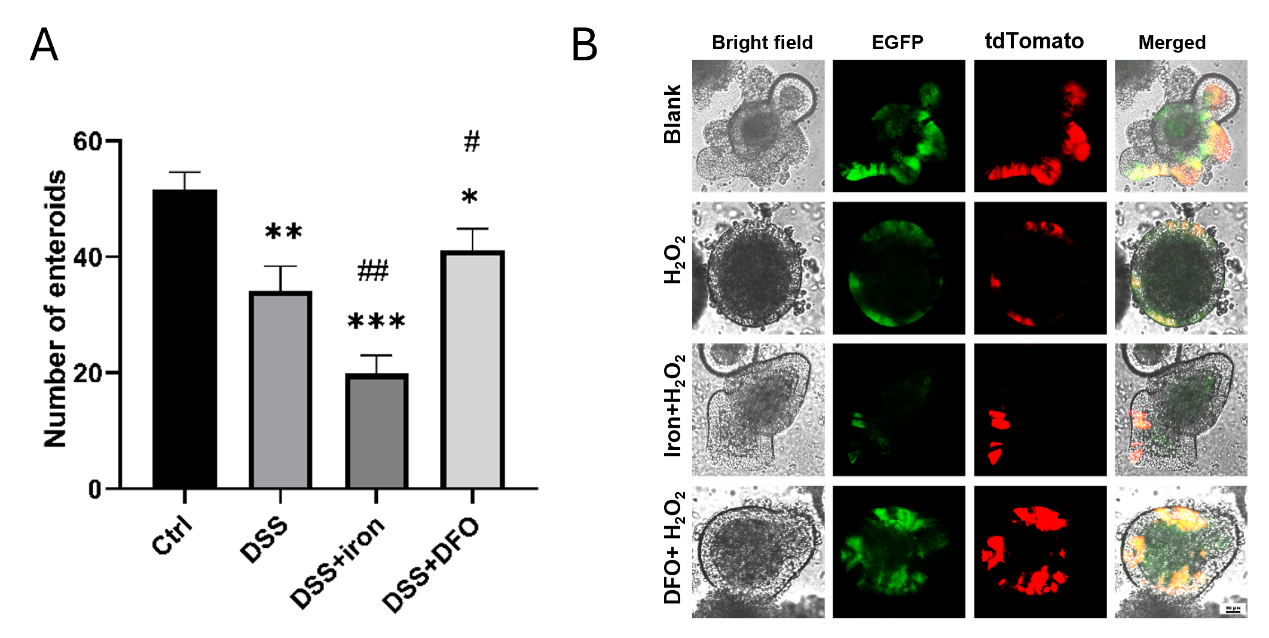


**Fig. S3. Iron content impaired the formation and growth of intestinal organoids.** (A) Statistical analysis for the number of enteroids cultured for 5 days using small intestinal isolated from different groups. (B) lineage tracing of EGFP positive Lgr5^+^ ISCs and their tdTomato^+^ progenies treated by iron or DFO in the presence of H_2_O_2_. *: *P*< 0.05, **: *P*< 0.01, ***: *P*< 0.001, compared to control group or blank group. #: *P*< 0.05, ###: *P*< 0.001, compared to DSS group.

**Fig. S4**


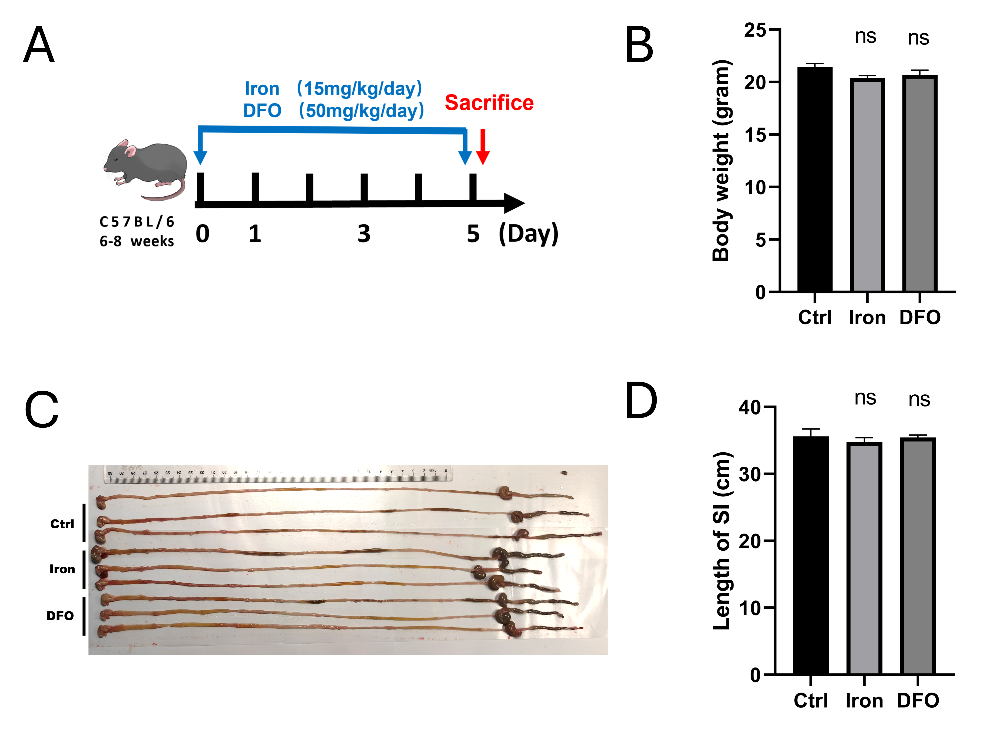


**Fig. S4. Iron supplementation and depletion at the current dose showed no obvious influences in healthy mice without DSS-induced colitis.** (A) Schematic diagram for the intervention of iron content in normal healthy C57BL/6J mice. (B) Comparison of body weight of different groups before sacrifice (n=3 each group). (C) Images of the whole gastrointestinal tract of each group (n=3 per group). (D) The comparison for the length of small intestines in different groups (n=3 per group). ns: Not significant (P >0.05).


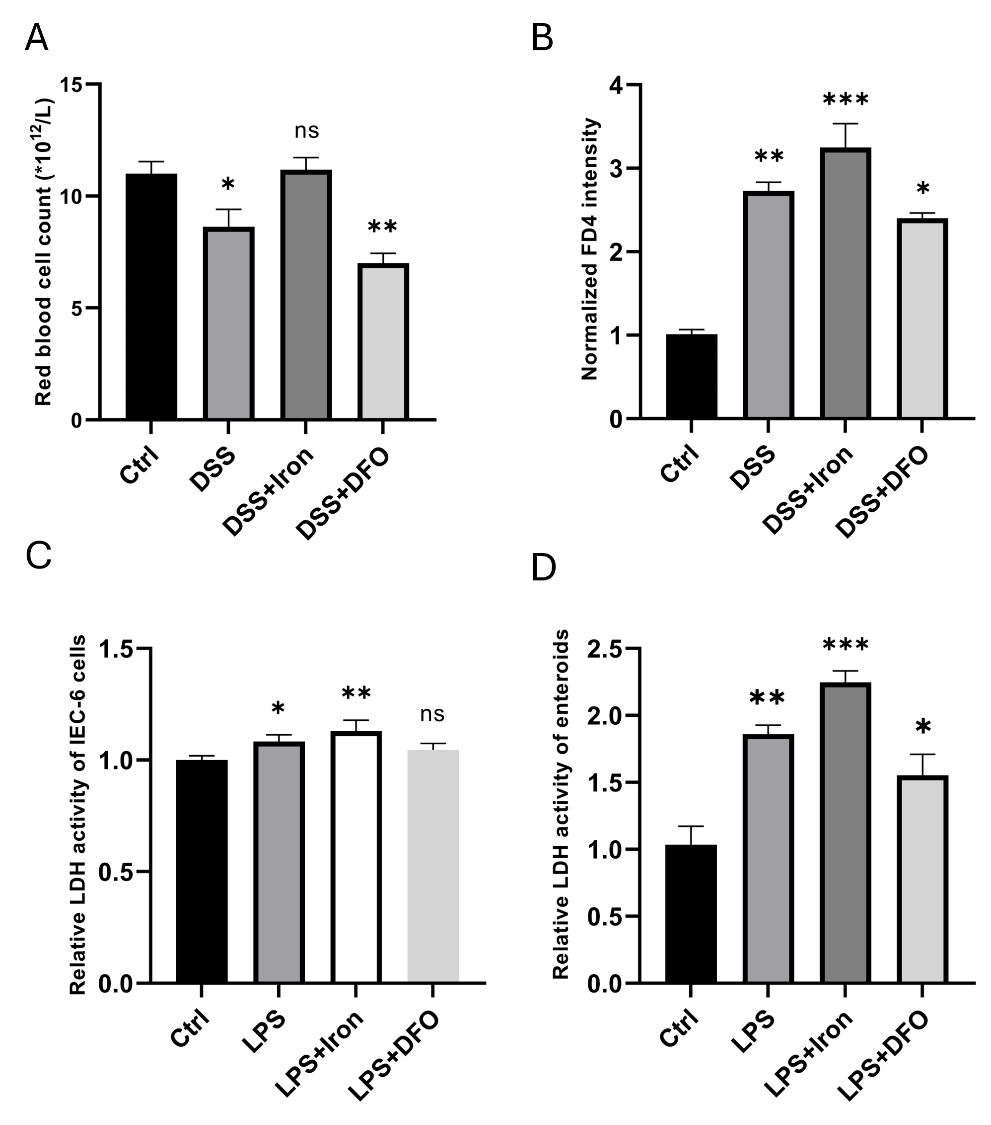
 **Fig. S5.**

**Fig. S5. Iron modulation affected RBC levels and intestinal permeability during DSS-induced colitis and cell viability in LPS-treated IEC-6 and enteroids.** (A) Red blood cells (RBC) count after continuous administration of DSS in drinking water (n=4 each group). (B) Normalized FITC-Dextran (FD4) levels in the serum samples from different groups (n=4 mice per group). (C) LDH assay showed that LPS induced apparent cytotoxicity in IEC-6 cells. Iron supplementation aggravated the toxicity, and DFO alleviated such cellular toxicity. (D) LDH assay using intestinal organoids. LPS induced serious cytotoxicity on enteroids, and iron modulation differently impaired such cytotoxicity. *: *P*< 0.05, **: *P*< 0.01, ***: *P*< 0.001, n.s: not significant, compared to control group.
